# Fatal Yellow Fever Vaccine–Associated Viscerotropic Disease — Oregon, September 2014

**Published:** 2015-03-20

**Authors:** Malini DeSilva, Arun Sharma, Erin Staples, Byron Arndt, Wun-Ju Shieh, Jim Shames, Paul Cieslak

**Affiliations:** 1 Epidemic Intelligence Service, CDC; 2 Neurocritical Care/Critical Care Medicine, Asante Health System, Medford, Oregon; 3 Arboviral Diseases Branch, Division of Vector-Borne Diseases, CDC; 4 Vista Pathology, Medford, Oregon; 5 Infectious Diseases Pathology Branch, Division of High-Consequence Pathogens and Pathology, CDC; 6 Jackson County Health and Human Services, Medford, Oregon; 7 Acute and Communicable Diseases, Center for Public Health Practice, Oregon Health Authority, Portland, Oregon

In September 2014, a previously healthy Oregon woman in her 60s went to a hospital emergency department with malaise, dyspnea, vomiting, and diarrhea of 3–5 days’ duration. She reported no recent travel, ill contacts, or dietary changes. Six days earlier, she had received a single dose of yellow fever vaccine and typhoid vaccine before planned travel to South America.

In the emergency department, the woman was afebrile but tachycardic and weak. Initial laboratory reports included a white blood cell count of 4,400/*μ*L (reference range [RR] = 4,800–10,800/*μ*L), platelet count of 84,000/*μ*L (RR = 150,000–400,000/*μ*L), potassium level of 2.8 mmol/L (RR = 3.5–5.1 mmol/L), and calcium level of 8.0 mg/dL (RR = 8.6–10.0 mg/dL). She was admitted to the hospital with diagnoses of gastroenteritis, malaise, dyspnea, and thrombocytopenia. Within 10 hours of admission, she experienced acute respiratory failure requiring intubation and mechanical ventilation. Contrast chest computed tomography indicated a substantial mediastinal mass. The patient experienced cardiogenic shock and acute renal failure and died 3 days after admission. At autopsy, the thymus was diffusely enlarged, consistent with thymoma. The concentration of acetylcholine receptor binding antibody in blood collected 1 day before death was 0.88 nmol/L (RR = ≤0.02 nmol/L), indicative of myasthenia gravis.

Tissue and serum samples were tested at CDC for evidence of yellow fever vaccine–associated viscerotropic disease (YEL-AVD), a serious adverse reaction resulting from the uncontrolled replication of vaccine virus and characterized by multisystem organ dysfunction; 60% of reported cases are fatal (1). Immunohistochemical staining indicated yellow fever virus antigen in tissue samples from various organs (Figure). Reverse transcription–polymerase chain reaction detected yellow fever vaccine viral RNA in multiple organs and in a serum sample that had been collected 2 days before death. Additionally, a serum sample obtained 1 day before death demonstrated evidence of yellow fever immunoglobulin M, with a yellow fever virus–specific neutralizing antibody titer of 640. Testing of yellow fever viral RNA from the vaccine lot used to vaccinate the woman identified sequences consistent with known vaccine strains without any notable mutations.

The patient’s clinical course and laboratory results, including her requirement for mechanical ventilation, platelets <100,000/*μ*L, hypotension requiring vasopressor drugs to maintain systolic blood pressure, and increase in creatinine to ≥1.5 times the upper limit of normal, met Level 1 diagnostic certainty for viscerotropic disease. The temporal relationship between yellow fever vaccination and development of symptoms was consistent with YEL-AVD (1). The presence of yellow fever virus–specific antigen in multiple organs demonstrated by immunohistochemistry, in addition to amplification of yellow fever 17D viral RNA from tissue, met criteria for definite yellow fever vaccine–associated causality (1). Both her age at vaccination and occult thymic disease likely predisposed this patient to YEL-AVD development.

The risk for YEL-AVD in the United States is approximately 0.4 cases per 100,000 doses of yellow fever vaccine distributed; older age and thymic disease have been associated with an increased risk for YEL-AVD (2). Risk increases to one case per 100,000 doses of yellow fever vaccine distributed for travelers aged ≥60 years and 2.3 cases per 100,000 doses for those aged ≥70 years (3). Of the first 23 YEL-AVD cases described, four (17%) were in patients who had a history of thymoma (4). Risk related to thymic disease might persist even after thymus resection (4). The incidence of thymoma in the United States is approximately 0.13 per 100,000 person-years, increasing with age and peaking among persons aged ≥60 years. Approximately one third of thymomas are diagnosed among asymptomatic patients on the basis of abnormal chest radiographs or computed tomography (5); 10%–20% of patients with myasthenia gravis have a thymoma, and approximately 30% of patients with thymoma have thymoma-associated myasthenia gravis (6).

Although yellow fever vaccination would have been contraindicated in this patient had it been known that she had a thymoma or myasthenia gravis, there is no national recommendation for routine screening for thymic disease before receipt of yellow fever vaccine. This appears to be the first published report on a case of YEL-AVD in a person with undiagnosed thymoma since the package insert for yellow fever vaccine available in the United States was updated to include a history of thymus disorder as a contraindication to vaccine administration in 2003 (2).

Although most persons have no or mild adverse events after yellow fever vaccination, the benefits of vaccination among travelers who have a limited exposure period need to be weighed against risk for adverse events (2,7). Yellow fever can range in severity from a mild febrile illness to severe disease with jaundice and hemorrhage; the case-fatality ratio for severe yellow fever disease is 20%–50% (7). An estimated 200,000 yellow fever cases occur worldwide annually, with approximately 87% in Africa (2). The live, attenuated vaccine is recommended for persons living in or traveling to tropical South America and sub-Saharan Africa (1); proof of yellow fever vaccination can be required for entry into certain countries. When determining whether a patient should receive yellow fever vaccine, the patient and clinician should discuss the risk for travel-associated yellow fever disease as indicated by season, destinations and duration of travel, likelihood of exposure to mosquitoes while traveling, and vaccination status, and weigh them against risks associated with vaccination.

## Figures and Tables

**FIGURE:**
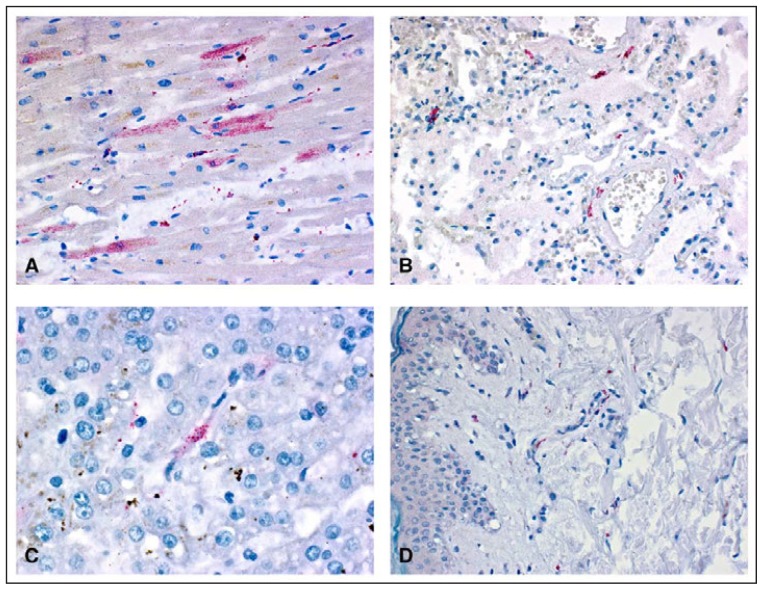
Yellow fever virus antigens (red) detected after immunohistochemical staining in tissue samples from various organs* of a patient who died from yellow fever vaccine–associated viscerotropic disease — Oregon, September 2014 * Sample A: myocytes in heart; sample B: fibroblasts in vascular wall in lung; sample C: kupffer cell in liver; sample D: fibroblasts and histiocytes in skin. (Immunoalkaline phosphatase with naphthol fast-red substrate and hematoxylin counterstain. Original magnifications: A = ×400; B = ×100; C = ×400; D = ×100.)
